# Nanodiamonds protect skin from ultraviolet B-induced damage in mice

**DOI:** 10.1186/s12951-015-0094-4

**Published:** 2015-05-07

**Authors:** Meng-Si Wu, Der-Shan Sun, Yu-Chung Lin, Chia-Liang Cheng, Shih-Che Hung, Po-Kong Chen, Jen-Hung Yang, Hsin-Hou Chang

**Affiliations:** Division of Plastic Surgery, Department of Surgery, Buddhist Tzu Chi General Hospital, No. 707 Sec. 3, Chung-Yang Rd, Hualien City, Hualien County 970 Taiwan; Department of Molecular Biology and Human Genetics, Tzu-Chi University, No. 701 Sec. 3, Chung-Yang Rd, Hualien City, Hualien County 970 Taiwan; Research Center of Nanobiomedical Science, Tzu-Chi University, No. 701 Sec. 3, Chung-Yang Rd, Hualien City, Hualien County 970 Taiwan; Department of Physics, National Dong Hwa University, No. 1 Sec. 2, University Road, Shoufeng Township, Hualien County 974 Taiwan; Nanotechnology Research Center, National Dong Hwa University, No. 1 Sec. 2, University Road, Shoufeng Township, Hualien County 974 Taiwan; Department of Biochemistry, School of Medicine, Tzu Chi University, No. 701 Sec. 3, Chung-Yang Rd, Hualien City, Hualien County 970 Taiwan; Institute of Medical Sciences, School of Medicine, Tzu Chi University, No. 701 Sec. 3, Chung-Yang Rd, Hualien City, Hualien County 970 Taiwan; Department of Dermatology, Buddhist Tzu Chi General Hospital, No. 707 Sec. 3, Chung-Yang Rd, Hualien City, Hualien County 970 Taiwan

**Keywords:** Nanodiamonds, Ultraviolet, Sunburn, Sunscreen

## Abstract

**Background:**

Solar ultraviolet (UV) radiation causes various deleterious effects, and UV blockage is recommended for avoiding sunburn. Nanosized titanium dioxide and zinc oxide offer effective protection and enhance cosmetic appearance but entail health concerns regarding their photocatalytic activity, which generates reactive oxygen species. These concerns are absent in nanodiamonds (NDs). Among the UV wavelengths in sunlight, UVB irradiation primarily threatens human health.

**Results:**

The efficacy and safety of NDs in UVB protection were evaluated using cell cultures and mouse models. We determined that 2 mg/cm^2^ of NDs efficiently reduced over 95% of UVB radiation. Direct UVB exposure caused cell death of cultured keratinocyte, fibroblasts and skin damage in mice. By contrast, ND-shielding significantly protected the aforementioned pathogenic alterations in both cell cultures and mouse models.

**Conclusions:**

NDs are feasible and safe materials for preventing UVB-induced skin damage.

**Electronic supplementary material:**

The online version of this article (doi:10.1186/s12951-015-0094-4) contains supplementary material, which is available to authorized users.

## Background

All life forms on Earth are greatly influenced by solar energy (electromagnetic radiation), which includes ultraviolet (UV; 200–400 nm), visible (400–700 nm), and infrared radiation. UV radiation is classified as UVC (200–290 nm), UVB (290–320 nm), and UVA (320–400 nm), and these high-energy radiation can damage cells [1]. The ozone layer absorbs almost all UVC and a part of the UVB wavelengths, thus protecting against severe UV damage. UVA is less harmful, and UVB is the primary threat to human health, causing acute sunburn, photoaging, immunosuppression, and skin cancers [2,3]. Even brief exposure to UV can induce DNA damage, such as pyrimidine dimers [cyclobutane pyrimidine dimer and the (6–4) photoproducts], which can be carcinogenic in the absence of adequate reparative processes [4]. Moreover, in 1985, Farman et al. reported springtime ozone depletion (ozone hole) over the Antarctic region [5]. The World Health Organization (WHO) developed the UV Index (UVI) to quantify UV radiation; its daily forecasts are currently used in several countries for people to adopt adequate protective measures [6,7].

Using sunscreens to block UV and avoiding excessive solar exposure are recommended for preventing sunburns [8]. Macro-sized titanium dioxide (TiO_2_) and zinc oxide (ZnO) are conventional and safe sunscreen ingredients but have the disadvantages of uneven coverage and an opaque white appearance [9]. By contrast, nanosized TiO_2_ and ZnO provide more effective protection and acceptable cosmetic appearance and have been widely used in commercial sunscreens since the late 1990s [10]. However, health concerns regarding systemic absorption and reactive oxygen species (ROS) gradually increased [11]. Studies have shown no increased penetration of these nanoparticles (NPs) in intact skin [12,13], but it remains a concern in sunburned skin [14]. Moreover, minor contamination with anatase crystals of rutile nanosized TiO_2_ can elicit photocatalysis and induce cellular damage [15].

Currently, nanodiamonds (NDs) are widely investigated nanomaterials. Because of their nontoxicity and biocompatibility, they are especially suitable for biomedical applications such as drug delivery and bioimaging [16]. In addition, NDs attenuate UV radiation through absorption and scattering, a phenomena dependent on factors such as ND particle size and nitrogen defects [17]. Although NDs are theoretical sunscreen candidates according to absorption spectral studies, few studies have assessed their practical protective effects. Therefore, we investigate the efficiency and safety of NDs as a UV filter in both animal and cell culture models and compare the results with those of nanosized TiO_2_ and ZnO.

## Results

### UVB attenuation by nanosized ND-, TiO_2_-, and ZnO-coated films

**Figure 1:**
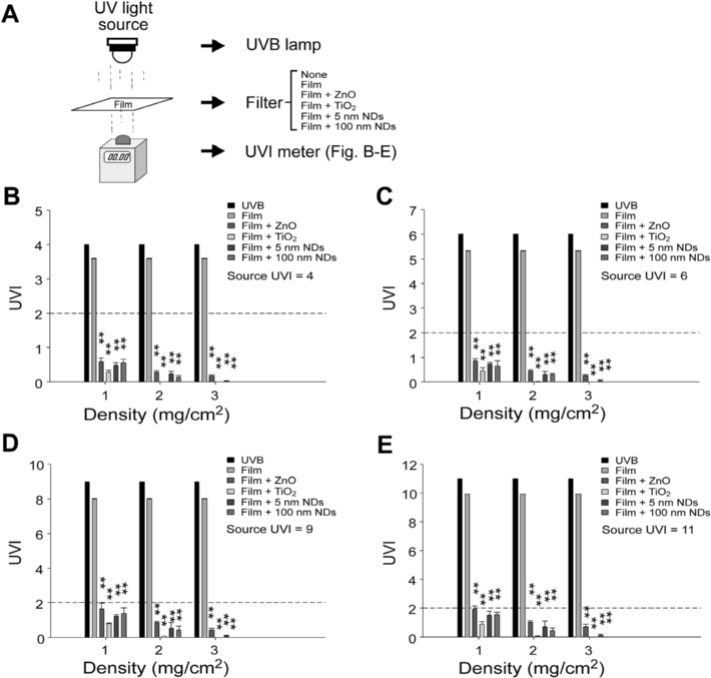
UVB attenuation by ND- and nanosized TiO_2_- and ZnO-coated films. Experiment setting **(A)**. Detected UVB UVI levels of the tested nanomaterial films at various nanomaterial concentrations. UVB at UVIs of 4 (100 mW/m^2^ UV_Ery_) **(B)**, 6 (150 mW/m^2^ UV_Ery_) **(C)**, 9 (225 mW/m^2^ UV_Ery_) **(D)**, and 11 (275 mW/m^2^ UV_Ery_) **(E)** were analyzed. The dashed line indicates a UVI of 2 (50 mW/m^2^ UV_Ery_), and UVB below this level is considered safe **(B–E)**. n = 3, *P < 0.05, **P < 0.01. Data are mean ± SD.

The UVB attenuation abilities of 5- and 100-nm NDs, nanosized TiO_2_, and ZnO and the association between nanomaterial concentration and UVB intensity were tested (Figure 1A, experiment setting). All four nanomaterials significantly reduced UVB intensity to a safe range (UVI < 2, Figure 1B-E) defined by WHO [6,7] even under the extreme UVB exposure of UVI 11 (Figure 1E). At a nanomaterial concentration of 2 mg/cm^2^, TiO_2_, ND, and ZnO exhibited approximately 99%, 94%, and 90% of UVB-blocking efficiencies, respectively. The efficiency of TiO_2_ was significantly higher than that of ZnO at all tested doses but higher than that of ND only at lower concentrations (1 and 2 mg/cm^2^) (Additional file 1: Figure S1). This experiment demonstrated ND efficiency in UVB attenuation.

### NDs protect cells from UVB damage

**Figure 2:**
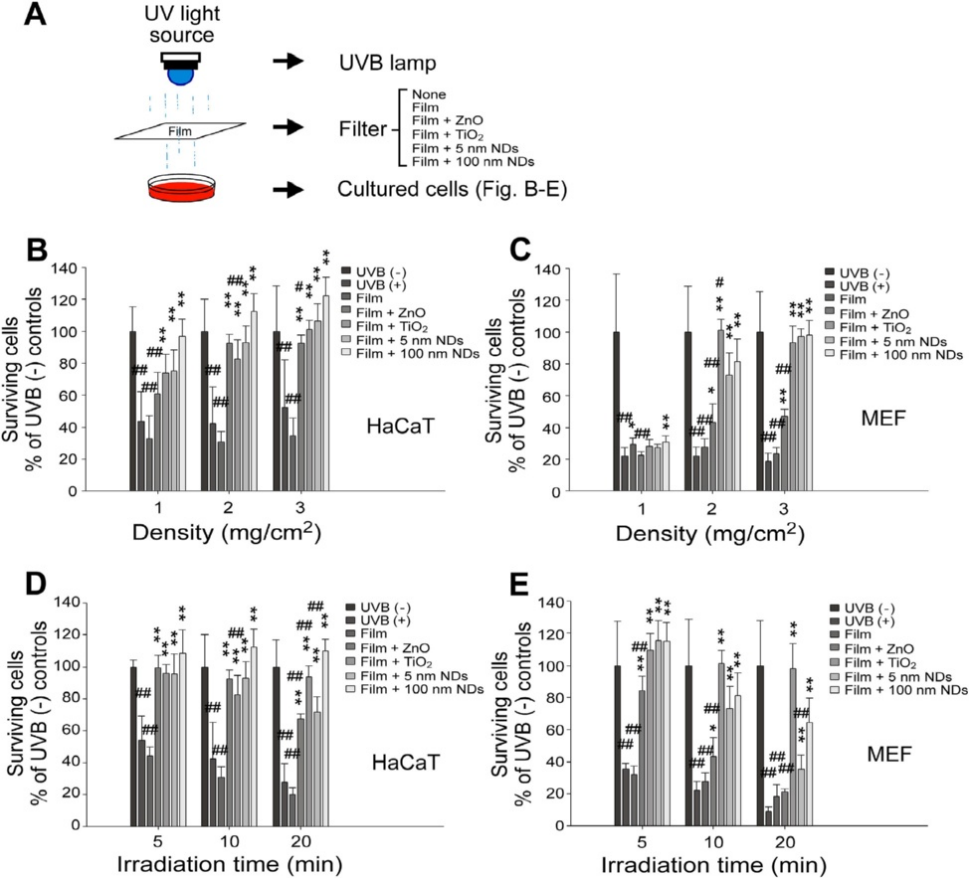
NDs protect cultured cells from UVB damage. Experiment setting **(A)**. Dose dependent **(B, C)** (UVI 6 for 10 min [68 mJ/cm^2^] at 1, 2, and 3 mg/cm^2^ density) and time-dependent **(D, E)** (UVI 6 for 5 and 10 min [34 and 68 mJ/cm^2^] at 2 mg/cm^2^ density) responses of nanomaterials in UVB protection of HaCaT **(B, D)** and MEF **(C, E)** cells were measured using the WST-1 assay. n = 6 (three experiments repeated twice). Groups without UVB exposure [UVB (−) control] was normalized to 100%. n = 6 (three experiments repeated twice); *P < 0.05, **P < 0.01, significantly better as compared with UVB (+) groups; ^#^ *P* < 0.05, ^##^ *P* < 0.01, significantly worse as compared with Film + 100-nm NDs” groups. Data are mean ± SD. The 10-min groups in **(D)** and **(E)** are adoptive from 2 mg/cm^2^ groups in **(B)** and **(C)**, respectively.

The protective efficiency of NDs was further investigated using a cell culture model. All four nanomaterials at medium–high concentrations (≥2 mg/cm^2^) protected human immortalized HaCaT keratinocytes and mouse embryonic fibroblasts (MEFs) from UVB damage (Figure 2). After UVB irradiation at a UVI of 6 for 10 min, all four tested nanomaterials (ZnO, TiO_2_, and 5- and 100 nm-NDs) offered considerable protection at all tested doses in HaCaT cell group, and the 100-nm NDs were the optimal materials for UVB irradiation shielding (Figure 2A, experiment setting; Figure 2B). By contrast, MEFs were more sensitive to UVB under the same conditions [Figure 2B–C; UVB (+) group survival in MEFs was lower than that in HaCaT cells]. All tested materials did not offer adequate protection at low concentrations (1 mg/cm^2^) (Figure 2C). The protective efficiency of all materials increased (TiO_2_ > NDs > ZnO) on increasing the concentration to 2 mg/cm^2^. No significant differences were observed between 5-nm and 100-nm ND groups, whose cell viability averaged 73% and 82%, respectively (Figure 2C). At 3 mg/cm^2^, the cell viability of ND groups increased and did not differ significantly from that of the TiO_2_ group. However, no clear improvement was noted in the ZnO group (Figure 2C). Untreated MEF cells attached normally and displayed fusiform appearance. By contrast, after direct UVB exposure with and without shielding with transparent plastic films, fewer cells attached and were surrounded by cellular debris (Additional file 1: Figure S2).

Next, the influence of irradiation time on cell viability was examined. In agreement with the dose experiments of the shielding materials, HaCaT keratinocytes displayed relatively higher resistance against UVB irradiation; at a concentration of 2 mg/cm^2^, all tested materials offered considerable protection after 5-, 10-, and 20-min UVB irradiations (Figure 2D). Because the protective efficiency of 100-nm ND was significantly higher than those of all other materials in the 20-min group (Figure 2D), it is the optimal material. By contrast, in MEF experiments, complete protection was observed only in the 5-min group; the efficiency of ZnO continued to be lower than those of the others (Figure 2E). The mechanism underlying 100-nm ND and TiO_2_ being optimal for HaCaT cell and MEF survival, respectively, is unclear. A possible explanation is the differential sensitivity of these two cells types in response to wavelengths after differential UVB absorption, penetration, and emission; this mechanism is worthy of further investigation. These results collectively indicate the protective efficiency of ND against UVB irradiation. In addition, 100-nm NDs exhibit enhanced performance compared with 5-nm NDs (Figure 2D–E, 20-min group). Consequently, we focused on 100-nm NDs in the subsequent *in vivo* experiments.

### NDs do not exhibit photocatalytic activity

Both ZnO and anatase form TiO_2_ exhibit UV-induced photocatalytic activities [18-20], which induces ROS and damages human cells. In addition, impurity doping leads to anatase TiO_2_ exhibiting photocatalytic activity under visible light irradiation [21-27]. Although rutile TiO_2_ is used in cosmetic applications, minor anatase crystal contamination may elicit photocatalysis and induce cellular damage. Therefore, the photocatalytic activities of the four tested materials were analyzed using methylene blue (MB) degradation experiments. Both 5- and 10-nm NDs exhibited no obvious photocatalytic activity compared with the negative control MB group (Additional file 1: Figure S3). By contrast, rutile and anatase TiO_2_ samples considerably degraded MB (Additional file 1: Figure S3; anatase TiO_2_ was the positive control). These results suggested that NDs do not exhibit UVB-inducible photocatalytic activities.

### NDs protect C57BL/6J mouse skin from UVB-induced inflammation

**Figure 3:**
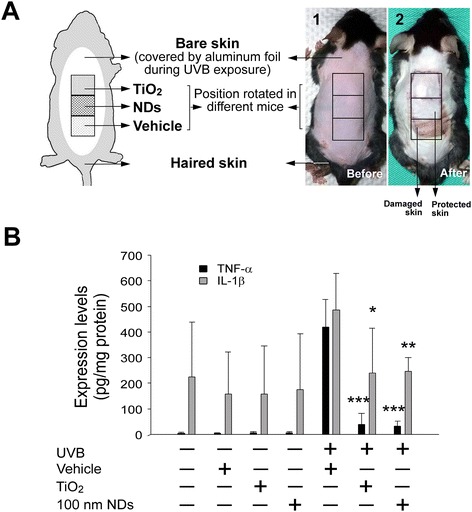
NDs protect C57BL/6J mouse skin from UVB-induced inflammation. Experiment setting **(A)** and the typical appearance of hair-removed mouse skin before (A1) and three days after UVB irradiation [UVI 6, 20 min per day, 3 cycles; (135.9 mJ/cm^2^/day × 3 days)], with or without protection at 2 mg/cm^2^ nanomaterial density (A2). Bare skin surrounding the experimental area was protected using aluminum foil. Each material was applied to the anterior, middle, and posterior position three times on three mice **(A)**. TNF-α and IL-1β in mouse skin are reported as pg per mg of protein **(B)**. n = 9 (three experiments repeated three times). *P < 0.05, **P < 0.01, ***P < 0.001; significant amelioration versus respective UVB vehicle groups. Data are mean ± SD.

The outermost strata of normal human skin are composed of multiple layers of dead corneocytes. Because the aforementioned cell culture model may not comprehensively reflect the complexity of human skin, an animal model was designed for studying the UVB-blocking efficiency of NDs. C57BL/6J mice were subjected to 20-min UVB irradiation at a UVI of 6 daily for three consecutive days; skin damage developed from the third day. An enzyme-linked immunosorbent assay (ELISA) detected elevation of both tumor necrosis factor-α (TNF-α) and interleukin-1β (IL-1β), two proinflammatory cytokines (Figure 3A, experiment setting; Figure 3B). The preliminary analysis showed that IL-1β increased earlier than TNF-α did and peaked 24 h after the second irradiation (Additional file 1: Figure S4A–S4B). Circulating TNF-α increased abruptly 24 h after the third irradiation, with the level corresponding to the severity of skin damage (Additional file 1: Figure S4B–S4D). To prevent skin damage outside the tested area (Additional file 1: Figure S4D), aluminum foil covered the nontested regions in subsequent experiments (Figure 3A). In placebo groups, materials such as vehicle, TiO_2_, and 100-nm NDs (2 mg/cm^2^) applied without UVB exposure were safe. By contrast, in mice receiving the aforementioned materials under the same conditions but irradiated with UVB at a UVI of 6, those covered with TiO_2_ and NDs showed only milder injuries compared with those covered with vehicle (Figure 3A-2). To further analyze the protective role of NDs in epidermal barrier functions, a dye exclusion experiment was employed, following methods modified from previous reports [28,29]. UVB irradiation damaged the epidermal barrier function of the experimental mice, thus causing higher levels of dye retention in the skin tissues (Additional file 1: Figure S5, vehicle groups). By contrast, TiO_2_- and 100-nm-ND-shielded skin samples tended to maintain a relatively undamaged epidermal barrier function, as indicated by the dye retention levels, which were lower than those in vehicle-shielded control groups (Additional file 1: Figure S5). Cytokine level changes were consistent with the skin damage results. In the experiment without UVB irradiation, nanosized TiO_2_ and 100-nm NDs did not induce TNF-α and IL-1β elevation (Figure 3B), suggesting that these materials are safe on mouse skin. After UVB exposure, both TNF-α and IL-1β in TiO_2_ and ND groups were significantly lower than those in vehicle groups, and no differences were observed between those of TiO_2_ and NDs. These results suggested that 100-nm NDs protect mouse skin from UVB damage at an efficiency comparable with that of nanosized TiO_2_ (Figure 3B).

### NDs ameliorate UVB-induced skin hyperplasia and leukocyte infiltration

**Figure 4:**
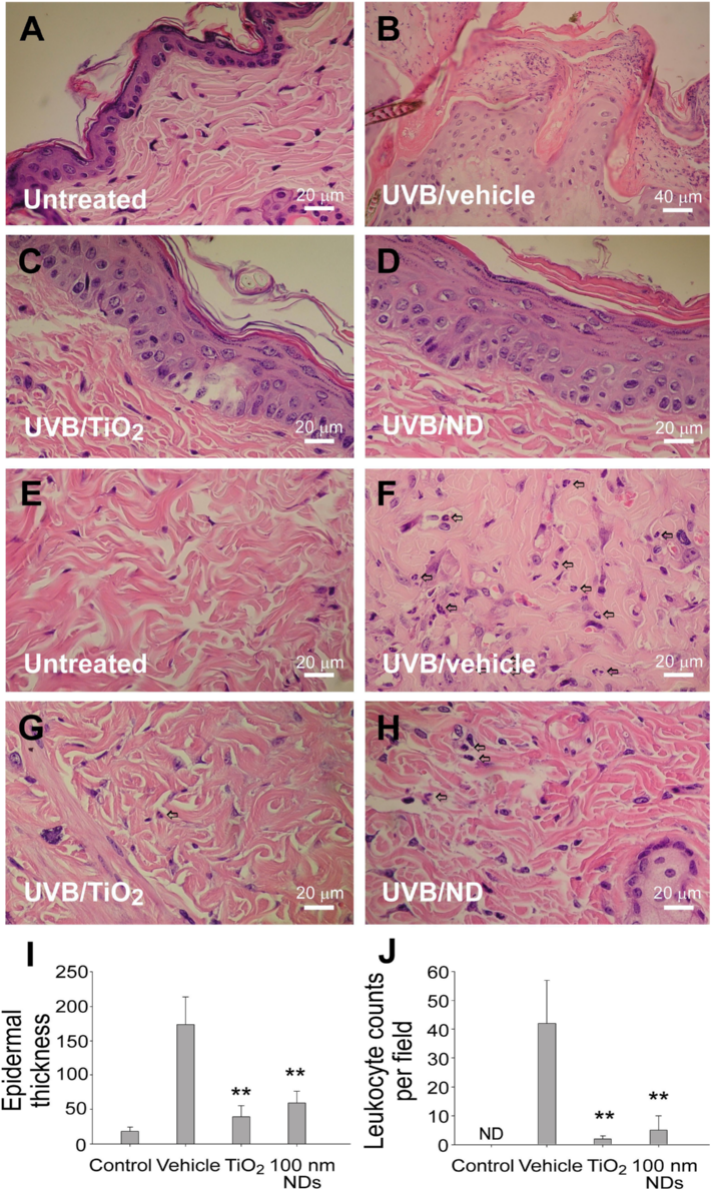
NDs ameliorate UVB-induced skin hyperplasia and leukocyte infiltration. Histological examinations of the epidermis **(A–D)** and dermis **(E–H)** revealed the alterations before **(A, E)** and 3 days after UVB irradiation [UVI 6, 20 min per day, three cycles; (135.9 mJ/cm^2^/day × 3 days)], with and without protection using 2 mg/cm^2^ TiO_2_ and 100-nm ND nanomaterials (**B–D, F–H;** H&E stain, × 400, scale bars in A and C–H = 20 μm and in B = 40 μm). The epidermal thickness was quantified as indicated **(I)**. The infiltrated leukocytes (indicated by arrows) were found in the dermis, especially in the vehicle group **(F–H)**; quantified results are presented in **(J)**. n = 9, **P < 0.01, significant amelioration versus vehicle groups. Data are mean ± SD.

Previous reports have indicated that hyperplasia of the strata granulosum and spinosum and increased leukocyte infiltration are involved in histological changes of UVB-irradiated skin [30,31]. In our study, considerable hyperplastic epidermis alterations were observed after UVB exposure of vehicle-applied mouse skin (Figure 4A vs. B). By contrast, the alterations in the TiO_2_ and 100-nm ND groups were much milder (Figure 4C and D vs. B; 4I, quantified results). The number of infiltrated leukocytes in the dermis was significantly higher in vehicle-treated groups than in the control groups (Figure 4E vs. F), clearly indicating an inflammatory response, but not in the nanosized TiO_2_ and 100-nm ND groups (Figure 4G and H vs. F; J, quantified results).

### Detections of 100-nm NDs on UVB-irradiated mouse skin and ND emission spectra

**Figure 5:**
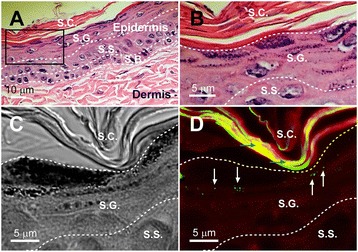
Detecting 100-nm NDs on UVB-irradiated mouse skin. An H&E-stained mouse skin section was used as a sample to illustrate the relative positions and morphology of the epidermis, dermis **(A)**, stratum corneum, and stratum granulosum (S.G.) **(B)**. The area indicated by the black box in **(A)** is magnified in **(B)**. Differential interference contrast **(C)** and confocal microscopy **(D)** images of a 100-nm-ND-shielded UVB-irradiated mouse skin. Dosages of UVB irradiation and 100-nm NDs are the same as those in Figure 4. White arrows indicate the fluorescent signals (501–511 nm; green labels) emitted by the 100-nm NDs, which did not penetrate beyond the S.G. **(D)**. Stratum corneum (S.C.), S.G., stratum spinosum (S.S.), and stratum basale (S.B.) are indicated. Blue arrows indicate keratin autofluorescence.

**Figure 6:**
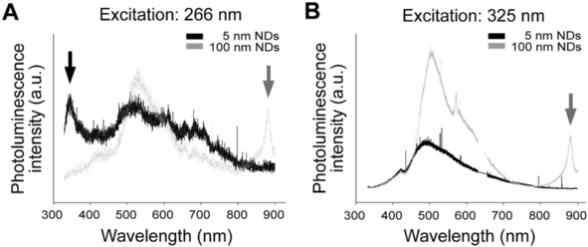
Photoluminescence of NDs. The photoluminescence properties of 5 and 100-nm NDs were characterized under excitation by 266 nm **(A)** and 325 nm UV **(B)** (i.e., the wavelengths approximately at the edges of UVB range), respectively. Gray arrows indicate the unique emission patterns of 100-nm NDs in the 800–900-nm (infrared) range **(A, B)**. A black arrow indicates the unique emission pattern of 5-nm NDs in the 320–400-nm (UVA) range **(A)**.

NDs are biocompatible materials [32]. However, their long-term effects remain unclear, and their accumulation and retention in the body should be avoided. To investigate whether 100-nm NDs can penetrate through the skin, particularly after sunburns, the skin of experimental mice were examined. We found 100-nm NDs only in the stratum granulosum and not in the deeper layers, even after intensive UVB irradiation (Figure 5). The epidermis, including the stratum granulosum, is gradually replacing by newly produced cells, and these NDs should theoretically along with the dead skin cells. Notably, on excitation with 266 nm UV, 5-nm but not 100-nm NDs displayed a unique emission pattern at approximately 320–400 nm in the UVA range (Figure 6A), which is harmful to the skin.

Furthermore, 100-nm but not 5-nm NDs elicited higher emissions in the infrared range on excitation with both 266- and 325-nm UV (Figure 6; 800–900-nm infrared). More UV energy can be transformed into relatively noncytotoxic infrared energy without eliciting harmful UVA, and this is likely part of the mechanism underlying 100-nm NDs exhibiting superior UV protection compared with 5-nm NDs (Figure 2C, 20-min group). These results collectively suggested that 100-nm NDs are a suitable sunscreen material.

## Discussion

UV-induced skin damage, such as acute sunburn, photoaging, and skin cancers, have been widely investigated through cell, animal, and human studies. However, few studies have studied the potential of NDs as UV filters. Our study found that NDs can attenuate UVB intensity, increase HaCaT and MEF cell viability, and ameliorate inflammatory responses of C57BL/6J mouse skin under UVB exposure. Moreover, their efficiency was comparable with those of nanosized TiO_2_ and ZnO, which are currently extensively utilized in sunscreens.

Irradiance (in W/cm^2^) is usually used to express UV intensity [1]. However, because the deleterious effects of UV are associated with UV wavelength, irradiance cannot reflect the true damaging ability. Erythemally weighted UV radiation (UV_Ery_) calculated according to the erythema reference action spectrum of the International Commission of Illumination more accurately estimates the harmful effects of UV on the skin [33]. This study adopted the UVI because it is based on UV_Ery_ and is the standard of UV reporting recommended by WHO, making our results applicable to everyday life [6]. Minimal erythema dose (MED) is another common measure of UV radiation [34,35]. However, because of the variance in individual sensitivity to UV radiation, MED is more appropriate for observational studies [1,36].

According to the regulations of the Food and Drug Administration of the United States, the sun protection factor of sunscreen ingredients are determined at a concentration of 2 mg/cm^2^ [37]. Therefore, we adopted the same concentration in our animal study. However, because most people apply insufficient sunscreen amounts, we further examined the protective ability of NDs at lower concentrations in UVB attenuation and cell viability tests.

Keratinocytes are a major cell type used in UV-related experiments because they are a major component of the epidermis, the outmost skin layer directly absorbing UV irradiation [15,38]. NDs, nanosized TiO_2_, and ZnO successfully attenuated extreme UVB (UVI = 11) to safe levels (UVI < 2). Additionally, these nanomaterials, particularly the 100-nm NDs, efficiently protected human HaCaT keratinocytes at 1 mg/cm^2^. Apart from keratinocytes, we used fibroblasts for *in vitro* analysis for the following three reasons. First, fibroblasts are the main cell type in the dermis, which constitutes a large percentage of the skin. UV with longer wavelengths penetrates deep into the dermal layer. Second, the UV-induced photodamage response and signaling pathways in humans and mice are not identical. Cutaneous IL-1β after UV irradiation arises from keratinocytes in humans but from infiltrating bone marrow-derived cells and Langerhans cells in mice [39]. Third, dermal fibroblasts are involved in photoaging and skin cancer [40,41]. Although MEFs are more sensitive to UVB irradiation than HaCaT cells, all the aforementioned nanomaterials efficiently protect MEFs at 2 mg/cm^2^.

Animal models are useful and essential in UV-induced skin damage studies. Because hairless mice develop squamous cell carcinoma after chronic UV exposure and support easy manipulation and skin observations [42], they have been widely utilized in skin-related research, especially SKH-1 mice [43,44]. We examined C57BL/6J mice because a previous study compared three strains of mice, C57BL/6J, SKH-1, and Balb/c, and concluded that C57BL/6J mice were the most similar to humans in photodamage, including in thickening of the epidermis, infiltration of inflammatory cells in the dermis, induction of TNF-α mRNA, and accumulation of glycosaminoglycans [45], whereas hairless SKH-1 mice lacked TNF-α mRNA induction. The histological findings were the same as those in the previous report, and a TNF-α assay correlated well with gross skin damage. Therefore, the C57BL/6J mouse model used in this study is effective for evaluating UV-induced injury.

The mechanism of nanosized TiO_2_ as a UV filter, which depends mainly on reflection, scattering, and absorption, has been studied extensively [9,46]. By contrast, ND–UV interaction has received little attention. The nitrogen-vacancy center in NDs absorbs strongly at approximately 560 nm and fluoresces efficiently at approximately 700 nm [47]. Moreover, ND optical properties can be altered through surface modification. For example, NDs covalently attached with octadecylamine emit bright blue light when irradiated with UV light [16]. Additionally, in this study, on excitation with UVB, 100-nm NDs elicited emissions not only in the visible-light range but also in the infrared range (Figure 6; 800–900 nm). These evidences collectively suggest that in addition to UVB shielding and scattering, NDs attenuate UVB energy by transformation it to safe visible and infrared light.

UV attenuation of nanomaterials depends largely on particle size. Primary nanosized TiO_2_ and ZnO particles cluster and form tightly bound aggregates with sizes between 30 and 150 nm. Subsequently, the aggregates loosen to form agglomerates with sizes over 1 μm. Nanosized TiO_2_ with an average size of 100 nm efficiently blocks both UVA and UVB. At an average size of 50 nm, UVB attenuation increases but UVA attenuation decreases. On reducing the average size to 20 nm, nanosized TiO_2_ offers significantly lower protection against both UVA and UVB [10]. Smaller, stabilized, and nonagglomerated TiO_2_ nanoparticles have superior UV attenuation [48]. NDs of various particle sizes have been studied and 100 nm NDs were suggested to be potentially useful in sunscreen formulations because of visual transparency and remarkable UVA, UVB, and UVC shielding [17].

## Conclusions

This study demonstrated for the first time that NDs attenuate UVB and efficiently protect keratinocytes, fibroblasts, and C57BL/6J mouse skin from UVB-induced damage. The 100-nm NDs exhibit superior UVB attenuation compared with nanosized TiO_2_, ZnO, and 5-nm NDs in the HaCaT keratinocyte model, and both 5 and 100-nm NDs exhibit superior UVB attenuation compared with nanosized ZnO in the MEF model. The protective efficiency of 100-nm NDs is comparable to that of nanosized rutile TiO_2_ in the animal model. Additionally, NDs are safe materials without considering the elicitation of ROS during UV irradiation. These results collectively suggest that NDs can be a “diamond-class” sunscreen ingredient.

## Methods

### UV related equipment and nanomaterials

UVB radiation was generated using a UVB lamp (G25T8E, Sankyo Denki Co., Kanagawa, Japan) with peak emission at 306 nm. UV intensity, reported as the UVI, was measured using a UVI meter (ARCS Precision Co., Taichung, Taiwan). Using previously reported methods [33,35], the conversion of UV-irradiation dose versus the UVI is as follows: 1 UVI = 25 mW/m^2^ UV_Ery_ (erythemally weighted UV radiation). Irradiation at a UVI of 6 for 20 min, used in our mouse experiments, were equivalent to 6 × 25 mW/m^2^ UV_Ery_ × 1200 s (=180 J/m^2^ UV_Ery_; = 1359 J/m^2^ UVB; = 135.9 mJ/cm^2^; = 3.775 MED; for B6 mice, 1 MED = 36 mJ/cm^2^ [35]). Rutile nanosized TiO_2_ was purchased from Advanced Ceramics Nanotech Co., Ltd (Tainan, Taiwan). A UV cut-off filter was fabricated by depositing the nanomaterials on a commercial food wrap film. A lubricating jelly (PDI, Orangeburg, NY, USA) composed of water and glycerin served as the vehicle for mixing the nanomaterials; it facilitated even dispersion of nanomaterials and its adherence to the film and skin.

### MEF and HaCaT keratinocyte cell cultures

MEFs were obtained using previously described methods [49,50]. Human immortalized HaCaT keratinocytes were maintained using previously described methods [51]. After thawing the frozen MEF and HaCaT cells, they were cultured with Dulbecco’s modified Eagle’s medium (DMEM) containing 10% fetal bovine serum (FBS), L-glutamine, and penicillin–streptomycin and grown in a 37°C, 5% CO_2_ incubator. The medium was changed after the first day and every two days thereafter. On confluence, the cells were trypsinized for passage.

### Animal study

Male C57BL/6J mice were purchased from the National Laboratory Animal Center and housed in the Laboratory Animal Center, Tzu-Chi University, until they were 8–9 weeks old. The hair on the backs of the mice was removed using commercial hair removal creams containing thioglycolate trihydrate (approximately 250 μL/mouse) 2–3 days before the experiments. The mice underwent the procedures under anesthesia with an intraperitoneal injection of ketamine : xylazine (80 : 10 mg/kg body weight). The research methods were approved by the Animal Care and Use Committee of Tzu-Chi University (approval ID 99047).

### Measurement of UV attenuation by nanoparticle-coated films

The attenuation efficiencies of four nanomaterials—5-nm NDs, 10-nm NDs, nanosized rutile TiO_2_, and ZnO—in attenuating UV radiation were tested. Each nanomaterial was mixed thoroughly with the vehicle (lubricating jelly) and applied to a thin plastic film at concentration of 1, 2, and 3 mg/cm^2^ for use as a UV filter. The UV intensity was measured using a UVI meter, and UVB at UVIs of 4, 6, 9, and 11, which respectively correspond to moderate, high, very high, and extreme exposure categories, were determined. The degree of attenuation was determined by comparing the UVI values with and without the UV filters.

### MB degradation

MB analyses for analyzing photocatalytic performance of TiO_2_ were conducted according to previously reported methods [52-54]. Photocatalytic efficiency was measured through the decomposition of 100 ppm (0.1 mg/mL) MB (Koch-Light Laboratories, Colnbrook, Bucks., England) at a nanomaterial concentration of 1.5 mg/mL. MB concentration intensity was monitored in the light absorption peak at a 663-nm wavelength by using a UV–Vis spectrometer. UV irradiation was performed using a UVB lamp (G25T8E, Sankyo Denki) with an average power density of 150 mW/m^2^ UV_Ery_ and 15 cm of spacing between the UV source and the sample. Anatase TiO_2_ (UV100, Sachtleben, Germany), which exhibits photocatalysis only when irradiated with UV light [24,55], served as the positive control in photocatalytic MB.

### Cell viability analyses

We used the cell culture model for evaluating the protective effects of the four nanomaterials. Cultured MEFs were seeded in a 96-well microplate (10^5^ cells/well) containing 200 μL/well of DMEM and 10% FBS, and grown in a 37°C, 5% CO_2_ incubator. On the second day, the MEFs were irradiated with UVB at a UVI of 6 for 5, 10, and 20 min with and without the UV filter containing 2 mg/cm^2^ of each nanomaterial. In addition, the cells were irradiated for 10 min with UV filters containing 1, 2 and 3 mg/cm^2^ of the nanomaterials. The culture medium was replaced by no-phenol-red medium during UVB irradiation. On the third day, the cultured cells were water-soluble tetrazolium (WST-1) assayed (Roche Diagnostics GmbH, Mannheim, Germany) [56,57]. The culture medium was removed and 100 μL of WST-1 reagent diluted with RPMI-1640 medium (1:19) was added to each well. After incubating at 37°C for 30 min, the WST-1 solutions were pipetted to a new microplate, and absorbance at 450 nm was measured using an ELISA reader. The cells not subjected to UVB irradiation were considered to have 100% cell survival, and the viability of the other study groups was calculated by comparing the WST-1 assay results.

### Cytokine analyses

We established an animal model to examine the efficiency of these nanomaterials in blocking UVB radiation. After irradiating the mice, TNF-α and IL-1β levels in the skin, which represents the degree of injury, were measured using ELISA (BioLegend, San Diego, CA, USA). The bare back skin of the mice was marked with four 1 cm × 1 cm areas on which were applied nothing, vehicle (jelly), 2 mg/cm^2^ 100-nm NDs, and nanosized TiO_2_. The mice were not subjected to UVB irradiation, and these materials were removed after 20 min. This procedure was repeated on three consecutive days. On the fourth day, the mice were sacrificed by CO_2_ inhalation and the skins were processed for a cytokine assay. In another group of mice, vehicle (jelly), 2 mg/cm^2^ 100-nm NDs, and nanosized TiO_2_ were applied in three longitudinally adjacent 1 cm × 1 cm areas on the back midline. The bare skin around the experimental area was protected with aluminum foil. The mice were irradiated with UVB at a UVI of 6 for 20 min, after which these materials were removed. This procedure was repeated on three consecutive days. The mice were sacrificed on the fourth day, and skin sections with a diameter of 0.7 cm containing the marked areas were excised for analysis. The skin samples were cut into small pieces and preserved in a 2-mL Eppendorf tube containing 700 μL of phosphate buffered saline (PBS) and 5 mM phenylmethanesulfonyl fluoride (PMSF), which decreased cytokine degradation [58]. The skin samples were homogenized and centrifuged at 4°C and 16,000 × g for 20 min. The supernatant was aspirated for additional quantification analyses of the protein and cytokine concentrations. The samples were placed on ice during processing [59,60].

### Enzyme-linked immunosorbent assay

TNF-α and IL-1β were quantified using commercial equipment (ELISA MAX™ Deluxe Sets, BioLegend, San Diego, CA, USA). One day prior to skin sample preparation, 100 μL of capture antibody solution was added to each well of a 96-well microplate and incubated overnight at 4°C. On the second day, after irradiation blocking, the solution was discarded and the microplate washed three times with 200 μL of PBS containing 0.05% Tween-20 per well. Two hundred microliters of assay diluent was added to the wells and then incubated at 37°C for 2 h. The microplate was washed again three times; and 100 μL/well solutions of serially diluted standards or processed skin samples were added to each well and then incubated overnight at 4°C. On the third day, the plate was washed three times and incubated at 37°C for 1 h after adding 100 μL of detection antibody to each well. Subsequently, 100 μL of avidin-horseradish peroxidase solution was added to each well and incubated at 37°C for 30 min. The plate was washed, 100 μL of tetramethylbenzidine substrate solution added to each well, and incubated at 37°C for 15 min. The reaction was stopped by adding 50 μL of 2 N hydrogen chloride solution to each well. Absorbance was measured at 450 nm by using an ELISA reader, and the standard curve was generated with a software program by using a four-parameter logistics curve-fitting algorithm. The cytokine levels in the skin samples were calculated from the standard curve. Because the UVB-damaged skin shrank and the skin sample may have been damaged during homogenization, the cytokine levels were normalized to the protein content for comparison. Dye reagent concentrate (Bio-Rad Laboratories, Hercules, CA, USA) was prepared by diluting (1:4) with distilled, deionized water. Bovine serum albumin with concentrations of 2, 1, 0.5, and 0.25 mg/mL were prepared and used as the protein standard. After thoroughly mixing 10 μL of each standard and sample solution with 190 μL of diluted dye reagent and incubating at room temperature for 5 min, the absorbance at 595 nm was measured using an ELISA reader.

### Histology examination

After the mice were sacrificed, the skin tissues were cut into three strips, preserved in 4% formaldehyde solution, dehydrated, and embedded in paraffin wax. Tissue sectioning and hematoxylin-eosin (H&E) staining were performed according to previously reported methods [57]. Thickness from the stratum granulosum to the stratum basale and neutrophil content within the dermis were measured at three random sites on each tissue strip.

### Confocal microscopy

Skin-tissue sections were obtained using protocols described in the aforementioned histology examinations. Fluorescence images of the skin sections were obtained using a confocal laser-scanning fluorescence microscope (TCS SP5, Leica, Germany; 63 × oil immersion). UV (405 nm) and argon lasers (458/476/488/514 nm) were employed for analyzing ND distribution in skin sections. ND fluorescence signals were excited at 405 nm and the resulting emissions were collected at 501–511 nm, as shown in green in Figure 5D. The fluorescence signals of the skin sections were excited at 488 nm and the resulting emissions were collected at 498–584 nm, as showed in red in Figure 5D.

### Photoluminescence

Different sizes of NDs (5- and 10-nm; Kay Diamond, USA) were used. For characterization, the obtained nanoparticle powders (NPs) was separately dispersed in deionized water at a concentration of 2 mg/mL. From each suspension, 20 μL was dropped onto a single crystal Si (100) wafer and dried. Photoluminescence (PL) of NPs were measured using a confocal microspectrometer (Jobin Yvon, T64000, France) equipped with a 325-nm liquid-N_2_ cooled charge-coupled detector (He–Cd gas phase laser, Kimmon Koha, Japan). The laser power supplied through the objective lens was estimated to be 20 mW (325-nm excitation, measured from the laser output). For macro-PL spectrometer (Horiba, HR-550, Japan) with a laser excitation wavelength of 266 nm (Nd–YAG, Laser-Export, USA), the laser power was 2 mW.

### Statistical analyses

The experimental results were analyzed using Microsoft Office Excel 2003 and SPSS 17, and the results reported as mean ± standard deviation (SD). Statistical significance of the obtained results was examined using a one-way analysis of variance the probability of type 1 error α = 0.05 was considered the threshold of statistical significance.

### Supporting information

Supporting information is available online.

## Additional files

Additional file 1: Figure S1. Relative UVB attenuation ability of tested materials. **Figure S2**. UVB illumination on mouse embryonic fibroblasts. **Figure S3**. Methylene blue degradation experiment. **Figure S4**. UVB-induced skin inflammation. **Figure S5**. Dye exclusion experiment.

## Abbreviations

UV: Ultraviolet
NDs: Nanodiamonds
WHO: World Health Organization
UVI: UV index
TiO_2_: Titanium dioxide
ZnO: Zinc oxide
ROS: Reactive oxygen species
NPs: Nanoparticles
MEFs: Mouse embryonic fibroblasts
ELISA: Enzyme-linked immunosorbent assay
TNF-α: Tumor necrosis factor-α
IL-1β: Interleukin-1β
UV_Ery_: Erythemally weighted UV radiation
CIE: International Commission of Illumination (usually abbreviated CIE for its French name, Commission internationale de l’éclairage)
MED: Minimal erythema dose
DMEM: Dulbecco’s modified Eagle’s medium
FBS: Fetal bovine serum
WST-1: Water soluble tetrazolium
PBS: Phosphate buffered saline
PMSF: Phenylmethanesulfonyl fluoride
HRP: Horseradish peroxidase
TMB: Tetramethylbenzidine
HCl: Hydrogen chloride
H&E: hematoxylin-eosin
SD: Standard deviation
ANOVA: Analysis of variance.
