# Supplementary material for: Nanodiamonds protect skin from ultraviolet B-induced damage in mice
Source: J Nanobiotechnology. 2015 May 7;13:35. doi: 10.1186/s12951-015-0094-4 (PMC4432518; doi:10.1186/s12951-015-0094-4)
Supplement: Additional file 1: Figure S1. — Relative UVB attenuation ability of tested materials. Figure S2. UVB illumination on mouse embryonic fibroblasts. Figure S3. Methylene blue degradation experiment. Figure S4. UVB-induced skin inflammation. Figure S5. Dye exclusion experiment. [file 12951_2015_94_MOESM1_ESM.docx]

**Supporting information for:**

**Nanodiamonds protect skin from ultraviolet B-induced damage in mice**

Meng-Si Wu,^1,2^ Der-Shan Sun,^2,7^ Yu-Chung Lin,^4^ Chia-Liang Cheng,^4,5^ Shih-Che Hung,^7^ Po-Kong Chen,^2^ Jen-Hung Yang^6,7,8^ and Hsin-Hou Chang^2,3,5,7^*

^1^ Division of Plastic Surgery, Department of Surgery, Buddhist Tzu Chi General Hospital. No. 707, Sec. 3, Chung-Yang Rd., Hualien City, Hualien County, 970, Taiwan

^2^ Department of Molecular Biology and Human Genetics, ^3^ Research Center of Nanobiomedical Science, Tzu-Chi University. No. 701, Sec. 3, Chung-Yang Rd., Hualien City, Hualien County, 970, Taiwan.

^4^ Department of Physics, ^5^ Nanotechnology Research Center, National Dong Hwa University. No. 1, Sec. 2, University Road, Shoufeng Township, Hualien County, 974, Taiwan

^6^ Department of Biochemistry, School of Medicine, Tzu Chi University. No. 701, Sec. 3, Chung-Yang Rd., Hualien City, Hualien County, 970, Taiwan.

^7^ Institute of Medicine, School of Medicine, Tzu Chi University. No. 701, Sec. 3, Chung-Yang Rd., Hualien City, Hualien County, 970, Taiwan.

^8^ Department of Dermatology, Buddhist Tzu Chi General Hospital. No. 707, Sec. 3, Chung-Yang Rd., Hualien City, Hualien County, 970, Taiwan

Page 1-5: Figure S1 - Figure S5

Page 6: Supplementary Methods and References

**Figure S1**


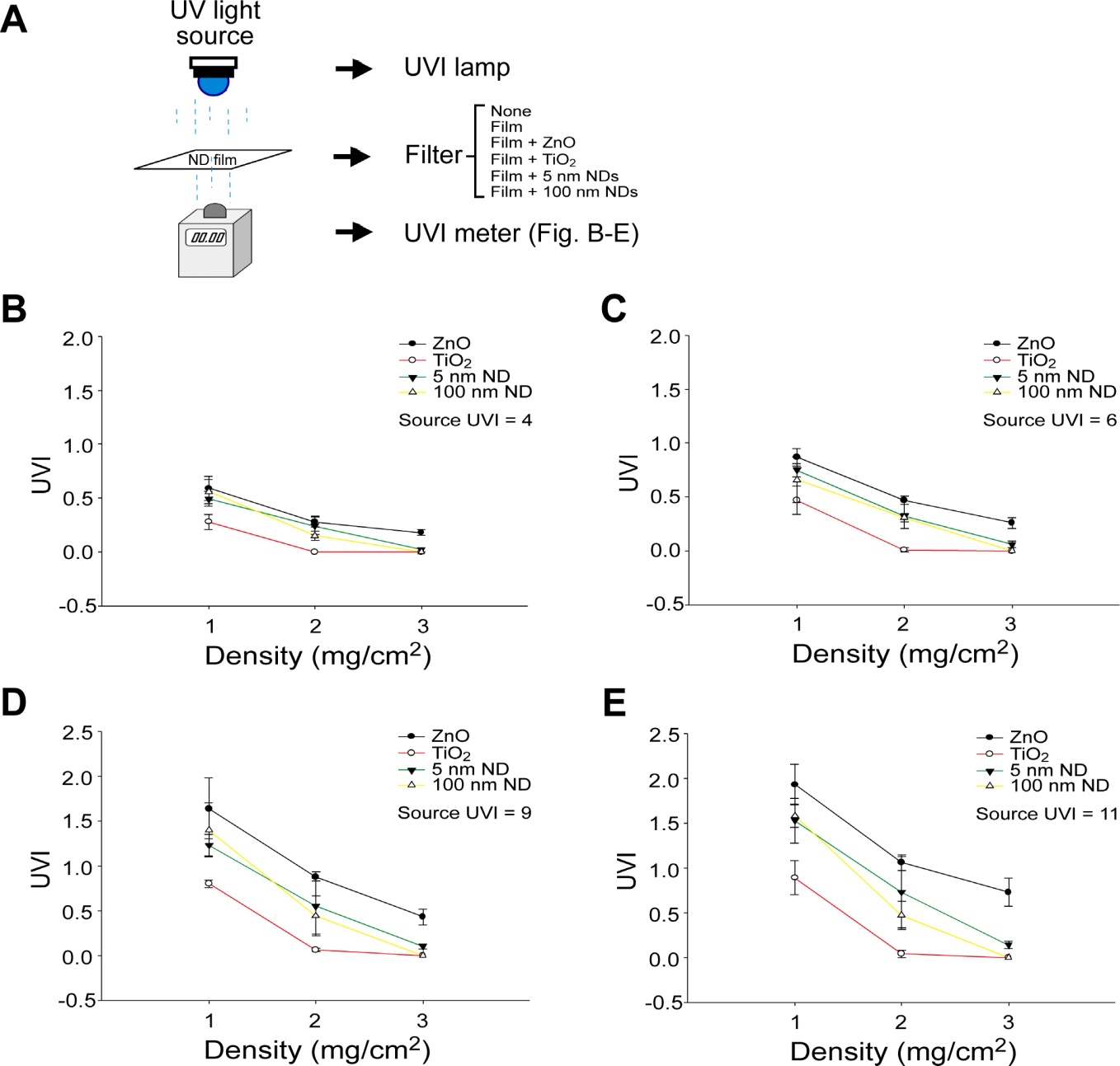


**Figure S1.** **Relative UVB attenuation ability of tested materials.** Graphs illustrated the relative UVB attenuation ability of ZnO, TiO_2_, 5 nm ND, 100 nm ND nanomaterials.

**Figure S2**


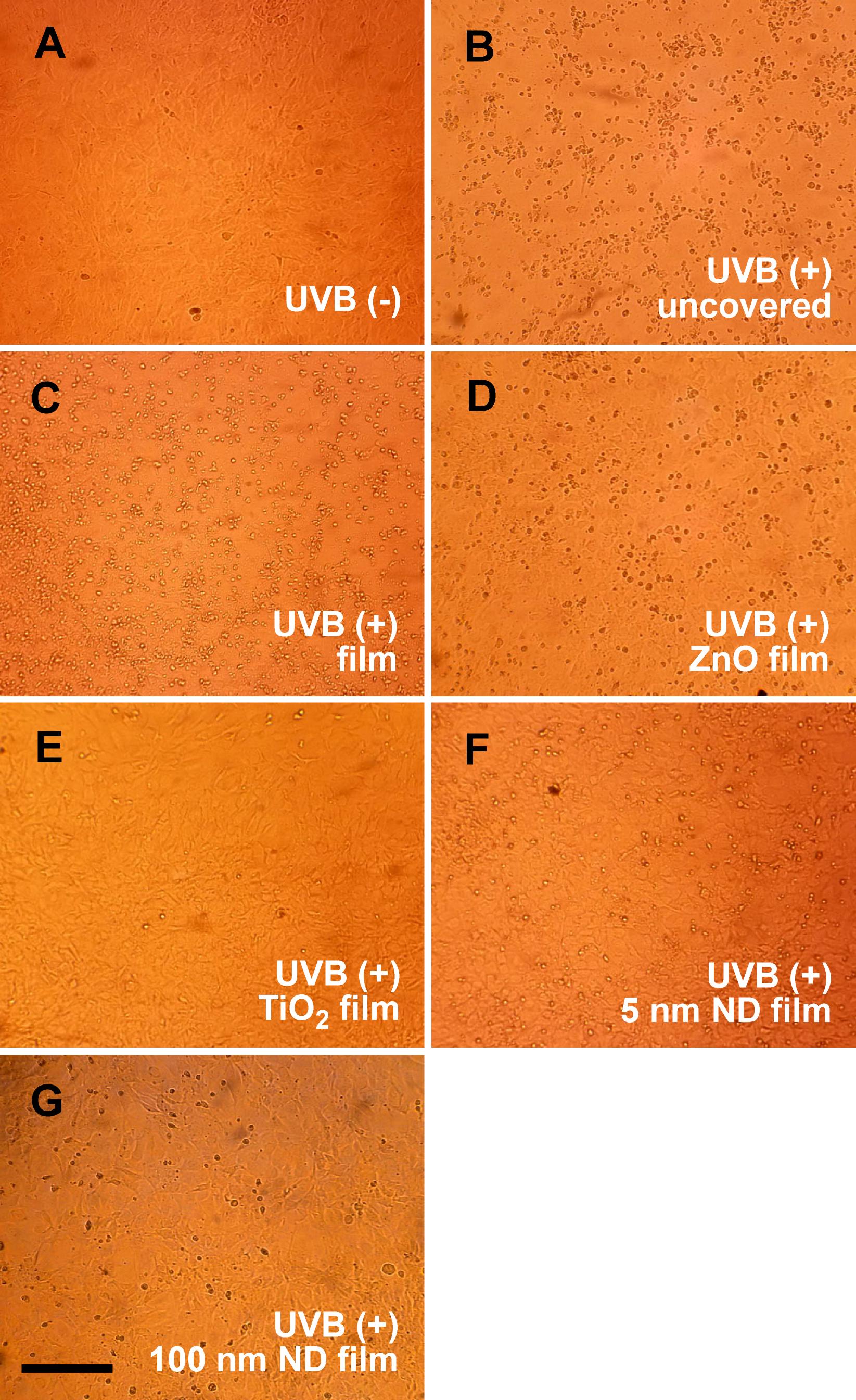


**Figure S2. UVB illumination on mouse embryonic fibroblasts (MEFs).** Cellular images of cultured MEFs before (A) and after (B-G) irradiation of UVB with or without protection by ZnO, TiO_2_, 5 nm ND, 100 nm ND nanomaterial-coated films (2 mg/cm^2^). (× 100, scale bar = 200 μm)

**Figure S3**


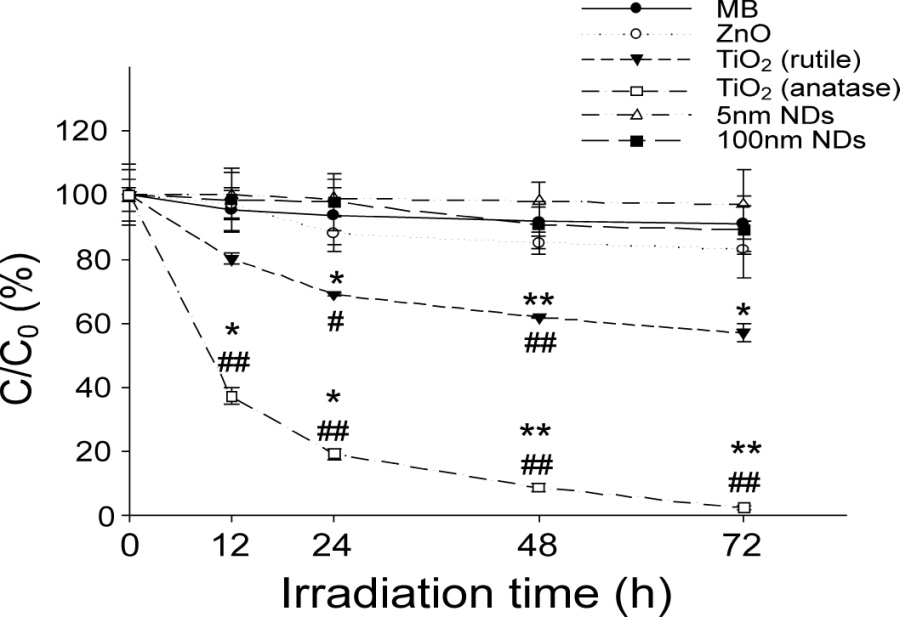


**Figure S3. Methylene blue (MB) degradation experiment.** To investigate the photocatalytic properties of nanoscaled materials, kinetic changes of MB degradation by UVB-irradiated ZnO, TiO_2_, 5 nm ND and 100 nm ND were analyzed. The MB levels without UVB irradiation were normalized to 100% (0 h groups). n = 3. * *P* < 0.05, ** *P* < 0.01 vs. MB groups; # *P* < 0.05, ## *P* < 0.01 vs. 100 nm ND groups.

**Figure S4**


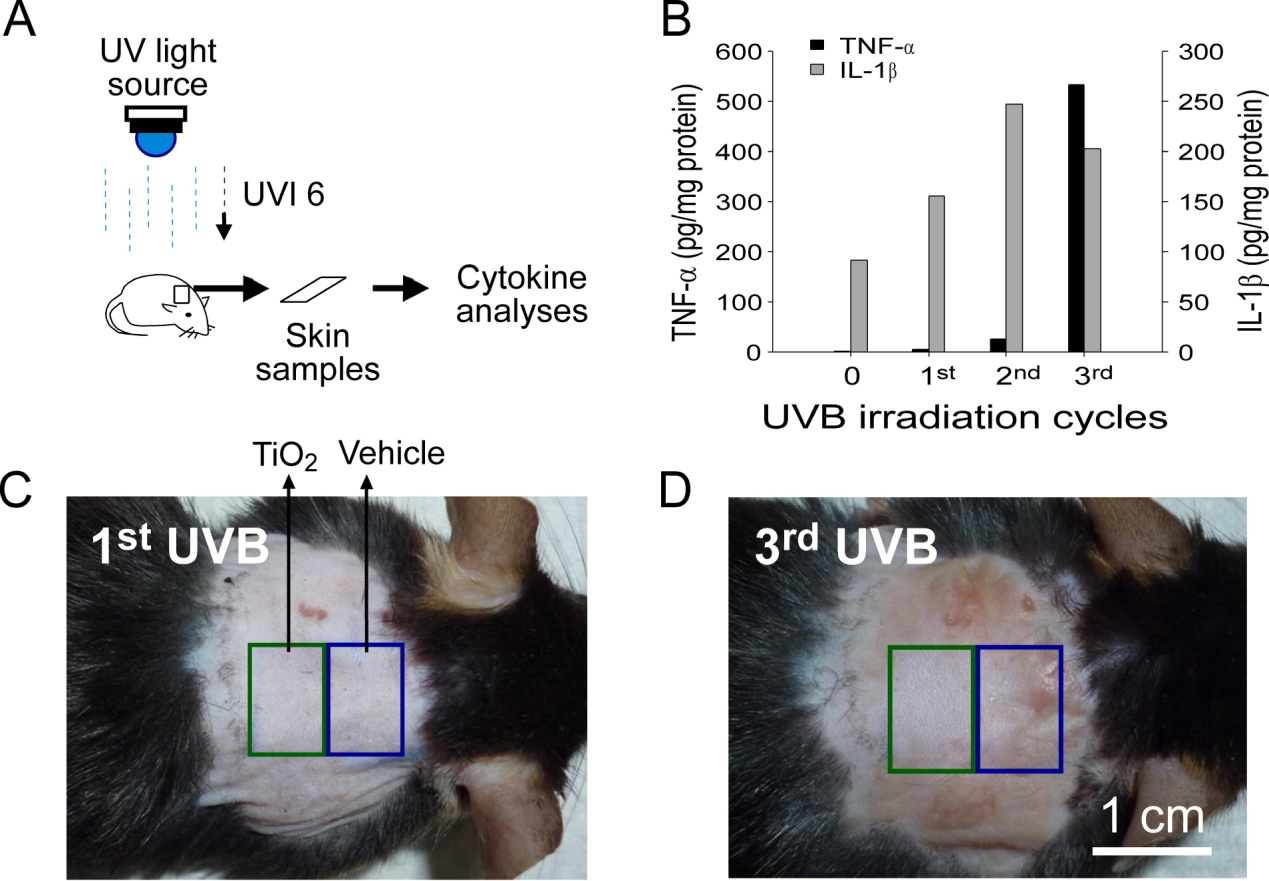


**Figure S4. UVB-induced skin inflammation.** The experiment setting (A). The levels of TNF-α and IL-1β in the mice skin after receiving one to three cycles of UVB irradiation without protection (B) (n = 2). The apperance of mouse skin damage 24 h after either one cycle (C) or three cycles (D) of UVB irradiation (1 cycle/24 h), with or without the protection by TiO_2_.

**Figure S5**


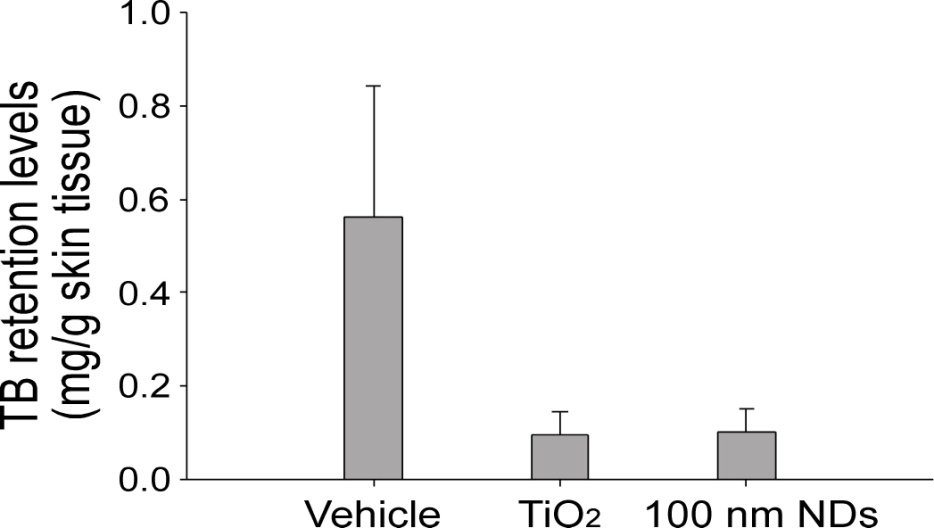


**Figure S5. Dye exclusion experiment.** To analyze the protective role of NDs in the epidermal barrier function, dye exclusion experiment was performed. The skin of hair-removed mice were exposured to UVB irradiation (UVI 6 for 40 min; 271.8 mJ/cm^2^). By 24 h later the mice were sacrificed and their UVB-irradiated skin portions were stained with toluidine blue (TB). Tissue retaining dye was extracted and then quantified using a UV-Vis spectrometer at 640 nm. Because the skin samples sheided by both materials tended to have less TB retention levels as compared to vehicle-sheided control groups, these results suggested that both TiO_2_ and 100 nm NDs contain UVB-protective effect on the epidermal barrier function.

**Supplementary Methods**

**Dye exclusion experiment**

To analyze the epidermal barrier function, a modified dye exclusion experiment was preformed according to previously reported methods [[1](#_ENREF_1), [2](#_ENREF_2)]. Vehicle (lubricating jelly; PDI, Orangeburg, NY, USA), 2 mg/cm^2^ 100-nm NDs, and nanosized TiO_2_ were applied on the backs of C57BL/6J mice in three longitudinally adjacent 1 × 1 cm areas. The mice were subjected to UVB irradiation (UVI 6 for 40 min; 271.8 mJ/cm^2^). Approximately 24 h later after UVB exposure, the mice were sacrificed and were rinsed in PBS and consecutively immersed in 25%, 50%, 75%, 100%, 75%, 50%, and 25% methanol for 1 min each. The mice were rehydrated in PBS and stained in 0.1% toluidine blue (Sigma-Aldrich, Inc., St. Louis, MO, USA) for 40 min and washed again in PBS. The skin samples were excised, weighed, and preserved in a 2-mL Eppendorf tube containing 700 μL of 50% methanol. Subsequently, the skin samples were homogenized and centrifuged at 16,000 × g for 20 min. The levels of skin-retained dye in the supernatant were quantified using a UV-Vis spectrometer at 640 nm.

**Supplementary References**

1. Segre JA, Bauer C, Fuchs E: **Klf4 is a transcription factor required for establishing the barrier function of the skin.** *Nat Genet* 1999, **22:**356-360.

2. Hardman MJ, Sisi P, Banbury DN, Byrne C: **Patterned acquisition of skin barrier function during development.** *Development* 1998, **125:**1541-1552.
